# Comparative Analysis of Selected Biochemical Markers Involved in Antioxidative, Immune Responses, and Thyroid Function in Wistar Rats Fed Diets Containing Baked Sprats

**DOI:** 10.3390/molecules31183169

**Published:** 2026-09-09

**Authors:** Urszula Pomietło, Ewelina Piasna-Słupecka, Sylwester Smoleń, Kinga Dziadek, Mariola Drozdowska, Ewa Piątkowska, Teresa Leszczyńska, Ivo Doskocil, Aneta Kopeć

**Affiliations:** 1 Department of Human Nutrition and Dietetics, Faculty of Food Technology, University of Agriculture in Krakow, 122 Balicka St., 30-149 Krakow, Poland; urszula.pomietlo@student.urk.edu.pl (U.P.); ewelina.piasna@urk.edu.pl (E.P.-S.); kinga.dziadek@urk.edu.pl (K.D.); mariola.drozdowska@urk.edu.pl (M.D.); ewa.piatkowska@urk.edu.pl (E.P.); teresa.leszczynska@urk.edu.pl (T.L.); 2 Laboratory of Mass Spectrometry, Faculty of Biotechnology and Horticulture, University of Agriculture in Kraków, 54 29 Listopada St., 31-425 Krakow, Poland; sylwester.smolen@urk.edu.pl; 3 Department of Microbiology, Nutrition and Dietetics, Faculty of Agrobiology, Food and Natural Resources, Czech University of Life Sciences Prague, Kamycka 129, Suchdol, 165 00 Prague, Czech Republic; doskocil@af.czu.cz

**Keywords:** sprats, rats, oxidative stress, thyroid function, minerals

## Abstract

Objective: This study was designed to evaluate the effect of baked freeze-dried sprats (SPR) added to experimental diets of Wistar rats, with induced streptozotocin (STZ) oxidative stress, on selected biochemical markers involved in antioxidative, immune responses, and thyroid function. Material and Methods: Male Wistar rats (n = 48) were assigned to four experimental groups: C control group, STZ group of rats injected with streptozotocin, SPR group fed diet containing sprats, and STZ + SPR group. Concentrations of selected minerals were determined in urine, feces and selected organs. Serum thyroid hormones (T3, T4), a thyroid-stimulating hormone (TSH) concentration, and the activity of antioxidant enzymes (GPx, GR, SOD) were assessed. The relative expression of *Txnrd1*, *Gpx1/3*, *Dio1–3* was quantified. Results: The highest concentrations of Mg, I, and Se were observed in the liver of the SPR and STZ + SPR groups compared with the C and STZ groups. T_4_ was significantly higher in all experimental groups compared to the C group. mRNA *Txnrd1* expression was higher in the thyroid of the SPR and STZ + SPR groups than in the C and STZ groups. The thyroid in STZ + SPR exhibited higher *Dio1* with lower *Dio2* and *Dio3* expression. Conclusions: It can be concluded that baked sprats are the source of bioavailable minerals involved in the reduction of oxidative stress and the modulation of iodine metabolism in rats with oxidative stress caused by impaired glucose metabolism.

## 1. Introduction

Sprats (*Sprattus sprattus*), fish belonging to the Clupeidae family, are rich not only in long-chain omega-3 polyunsaturated fatty acids (n-3 PUFAs), but also in different nutrients that are beneficial to the human organism. These fish, usually eaten whole with bones, are a great source of calcium, magnesium, zinc, selenium and iodine [1]. Their meat is rich in an easily assimilable, highly nutritional quality of protein [2,3]. Sprats eaten frequently can improve the nutritional status via the above-mentioned nutrients and strongly affect the immune response during oxidative stress and inflammation usually connected with infection. Nevertheless, fish intake in Poland remains at 13–14 kg per person per year, which is lower than the European average (23 kg/person/year) [4,5]. In populations with high fish consumption (e.g., ≥2 servings per week), a significant reduction in the risk of cardiovascular disease has been documented, on average by more than 8–50% depending on the study, whereas available evidence regarding effects on thyroid diseases remains inconclusive and requires further analyses [6,7].

According to recent global estimates of dietary inadequacy, the most prevalent shortfalls are for iodine and calcium, affecting approximately 68% and 66% of the global population, respectively [8]. Zinc also contributes substantially to the global burden of inadequacy; analyses based on data from 188 countries estimated the mean prevalence of inadequate zinc intake at ~17.3% of the population [9,10]. By comparison, a widespread global copper deficiency has not been reported; however, inadequate intake may affect a considerable proportion of certain populations, for example, more than 25% of adults in North America may not reach the Estimated Average Requirement (EAR; 0.7 mg/day) [11]. Insufficient selenium intake also remains a significant problem. Globally, it is estimated to affect approximately 0.5–1 billion people, with particularly high risk in selenium-poor regions such as Central Europe and central China [12,13]. Selenium deficiency causes lower synthesis of thyroid hormones [14,15]. Magnesium inadequacy has been reported in approximately 10–20% of the population in highly developed countries [16], whereas hypocalcemia often occurs as a secondary disturbance, resulting primarily from inadequate vitamin D_3_ and protein intake, which are essential for proper calcium homeostasis [17]. Collectively, these data underscore that inadequate micronutrient intake is not only a global issue but also has clear population-specific and sex-related dimensions. Iodine, selenium, magnesium, zinc, copper, and calcium remain among the most frequent insufficient micronutrients, and their adequate intake is essential for maintaining proper metabolic, immune, and endocrine function, but also protecting one from infection with viruses or pathogenic bacteria [18,19,20].

The nutritional state plays a very important role in appropriate defense, along with free radicals and the function of the immune system. During the SARS-CoV-2 pandemic, many articles focused on the properties of selected nutrients, especially minerals, with regards to the reduction of oxidative stress and inflammation. The nutritional status is strongly affected by eating habits and a well-balanced diet. An important part of a well-balanced diet is animal-origin food, rich in high quality protein, including meat, eggs, milk with dairy products and fish. Based on current recommendations, fish should be eaten at least twice per week, as it is rich in other nutrient minerals involved in the reduction of oxidative stress and inflammation [21,22].

Despite the growing number of studies on the role of individual micronutrients in regulating metabolic and immune processes, analyses focusing on the role of marine-derived foods are still limited. Existing reports have primarily addressed supplementation with iodine, selenium, or calcium, while substantially less attention has been devoted to small fish such as sprats, which may offer a unique combination of these elements, together with protein and omega-3 fatty acids [15,23,24]. In particular, little is known about their potential impact on the expression of genes related to micronutrient metabolism and immune responses [25,26]. Addressing this research gap is important not only from a scientific perspective but also from a practical standpoint, because in Central European populations, where fish intake remains low, increasing the share of small fish in the diet could represent an effective strategy to improve the intake of key immunomodulatory micronutrients.

The objective of this study was to evaluate the effect of adding baked freeze-dried sprats to experimental diets of Wistar rats with induced streptozotocin oxidative stress on immune system activity and thyroid function response.

## 2. Results

### 2.1. Chemical Composition of Sprats

**Figure 1:**
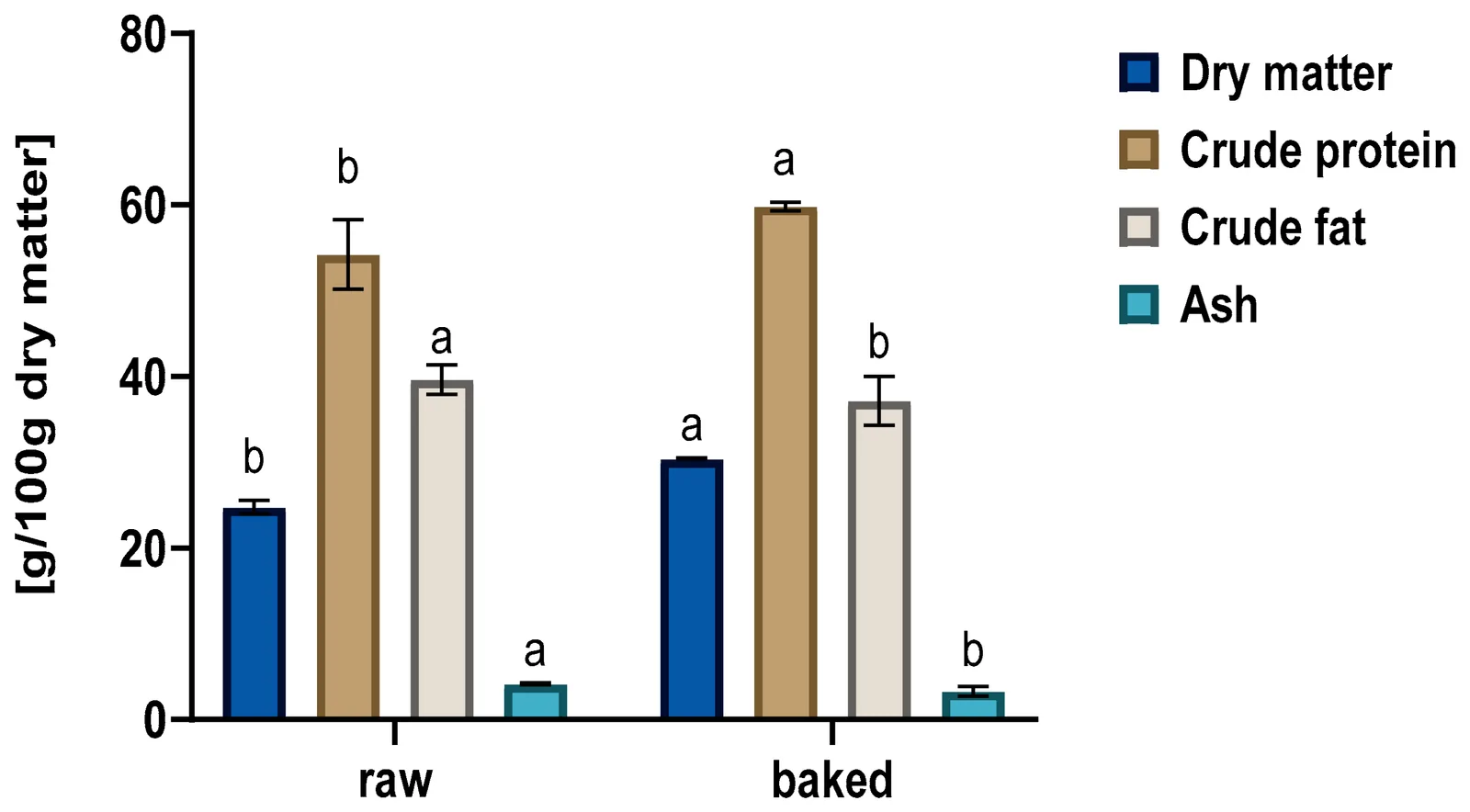
Chemical composition of sprats used in the experiment. Bars marked with different letters (a, b) differ significantly at *p* ≤ 0.05. [g/100 g dry matter].

Thermal processing markedly altered the chemical composition of sprats used in the rat feeding experiment. Compared with the raw variant, baked sprats exhibited higher dry matter and crude protein contents, along with lower crude fat and ash contents per 100 g of dry matter. These compositional differences were taken into account when formulating the experimental diets, ensuring balanced macronutrient profiles and energy contributions from the sprat addition (Figure 1).

### 2.2. Biological Value of Proteins

Apparent total digestibility (TD) of protein was significantly lower in the SPR group; no differences were observed in the apparent biological value (BV) or net protein utilization (NPU), indicating that the absorbed protein was utilized with efficiency comparable to that of the control diet (Table 1). Despite the higher fecal nitrogen content and fecal nitrogen excretion in the SPR group, nitrogen retention was significantly increased. The Hedges’ g analysis supported these observations by demonstrating a large effect size for TD and nitrogen retention, whereas only negligible effect sizes were observed for BV and NPU. It should be noted that the greater protein and nitrogen intake in the SPR group resulted from the higher amount of diet offered during the 5-day nitrogen balance period (70 g vs. 60 g in the C group).

### 2.3. Body Gain, Selected Organs Weight and Minerals Content in Urine, Feces, Kidney and Livers of Experimental Rats

Body weight gain was lower in STZ + SPR groups compared with the C group. No significant between-group differences were observed in liver, kidney or heart weights. (Table 2).

There were no significant changes in the urinary concentrations of magnesium (Mg), calcium (Ca), iron (Fe), and zinc (Zn) during the fifth week of the experiment. The highest concentrations of iodine (I), selenium (Se) and copper (Cu) were measured in the urine of the SPR group compared with the other experimental groups at this time point (Table 2). In the ninth week, urinary Ca and Mg levels were not significantly different. The concentration of Fe in urine significantly decreased in the STZ + SPR groups compared with the control (C) group. The concentrations of I and Cu were significantly lower in the STZ + SPR group than in the SPR group. Urinary Zn content was significantly lower in the SPR and STZ + SPR groups than in the C group in the ninth week. The Se concentration was significantly lower in the STZ + SPR group than in the STZ group in the ninth week of the experiment.

In both the fifth and ninth weeks, fecal Mg levels were significantly higher in the SPR and STZ + SPR groups than in the C and STZ groups (Table 3). The concentration of Ca in feces was significantly higher in the SPR and STZ + SPR groups than in the C and STZ groups during the fifth week. No significant changes were observed in fecal Fe, Se, Zn, Cu, or I concentrations in either the fifth or ninth week. Fecal Ca content did not differ among groups in the ninth week (Table 3).

The highest concentrations of Mg, I, and Se in the liver were observed in the SPR and STZ + SPR groups compared with the C and STZ groups. Ca and Fe levels were significantly higher in the C, SPR, and STZ + SPR groups compared with the STZ group. Zn and Cu contents were greatest in the STZ + SPR group compared with the other groups.

No significant differences were found in Ca and Mg concentrations in the kidney among groups. The highest Fe and I levels were recorded in the SPR and STZ + SPR groups compared with the C and STZ groups. Zn concentrations were highest in the kidney of both stress-induced groups (STZ and STZ + SPR). The highest Cu concentrations were found in the C, STZ, and STZ + SPR groups compared with the SPR group. Se content was highest in the STZ + SPR group compared with the others (Table 3).

### 2.4. Selected Biochemical Parameters in Serum of Experimental Rats

No significant differences were observed in the concentrations of TSH, iodotyrosine, or T3 among experimental groups (Table 4). The highest T4 concentration was detected in the STZ + SPR group compared with the SPR and C groups. T4 concentration was also significantly higher in the serum of rats from the group STZ + SPR compared to the serum of rodents from the SPZ group. TRIAC content was significantly higher in the C group than in STZ + SPR, whereas TETRAC concentration was highest in the STZ group compared with SPR. Salicylic acid (SA) significantly decreased in the serum of rats from the SPR group compared to the serum of rodents from the C group (Table 4).

The concentration of SeUrea was significantly higher in the serum of rodents in the STZ and STZ + SPR groups than in the serum of rats in the C and SPR groups.

No significant diet-related effects were observed for SeMetCys, SeVI, or iodide (I^−^) concentrations in the serum of experimental rodents. Selenomethionine (SeMet) concentration was significantly lower in the serum of the SPR group than in the STZ + SPR group. The highest level of Se2Cys was found in the serum of rats in the C group compared with the SPR and STZ + SPR groups. Glutathione reductase (GR) activity was highest in the serum of the C group compared with the serum of rodents in STZ and STZ + SPR. Glutathione peroxidase (GPx) activity was highest in the STZ + SPR and SPR groups. Superoxide dismutase (SOD) activity was highest in the STZ + SPR group compared with the C and STZ groups (Table 4).

No significant differences were observed in the serum concentrations of the analyzed cytokines among the experimental groups (Table 4).

### 2.5. Relative Genes and Protein Expression

The expression of the Gpx1 gene in the thyroid gland was significantly higher in the SPR group than in STZ + SPR. Gpx3 expression was highest in the STZ + SPR group compared with the expression of this gene in the thyroid gland of the rodents in the STZ group (Table 5). Txnrd1 expression was significantly higher in the thyroid gland of the SPR and STZ + SPR groups than in the C and STZ groups. Dio1 expression was highest in the thyroid gland of the STZ + SPR group compared to the thyroid glands of other experimental groups. Expression of Dio2 was significantly higher in the thyroid gland of the rodents in the C group compared to the expression of the groups SPR and STZ + SPR. Expression of Dio3 was significantly lower in the thyroid gland of the rodents in the STZ and STZ + SPR groups compared to the C group.

The expression of *Gpx1* was significantly lower in the liver of rodents in the groups STZ and SPR compared with the C and STZ+ SPR groups. The expression of *Txnrd1* was significantly higher in the liver of rodents in the STZ group than in the SPR group. Dio1 expression was significantly higher in the liver of the C group compared to the STZ group.

In the kidneys, expression of *Gpx3* was significantly higher in the C and STZ + SPR groups than in the STZ and STZ + SPR groups (Table 5). Various dietary treatments did not affect the expression of *Gpx1*, *Txnrd1*, *Txnrd2* and *Dio1* in the kidneys of experimental groups (Table 5).

In the lungs, the expression of *Gpx1* was significantly higher in the C and STZ groups compared to the SPR and STZ + SPR groups.

Expression of *Sod1* was significantly lower in the liver of STZ rats as compared to other experimental groups. In the heart, Sod1 expression was also significantly lover in the STZ group as compared to the C and SPR groups.

**Figure 2:**
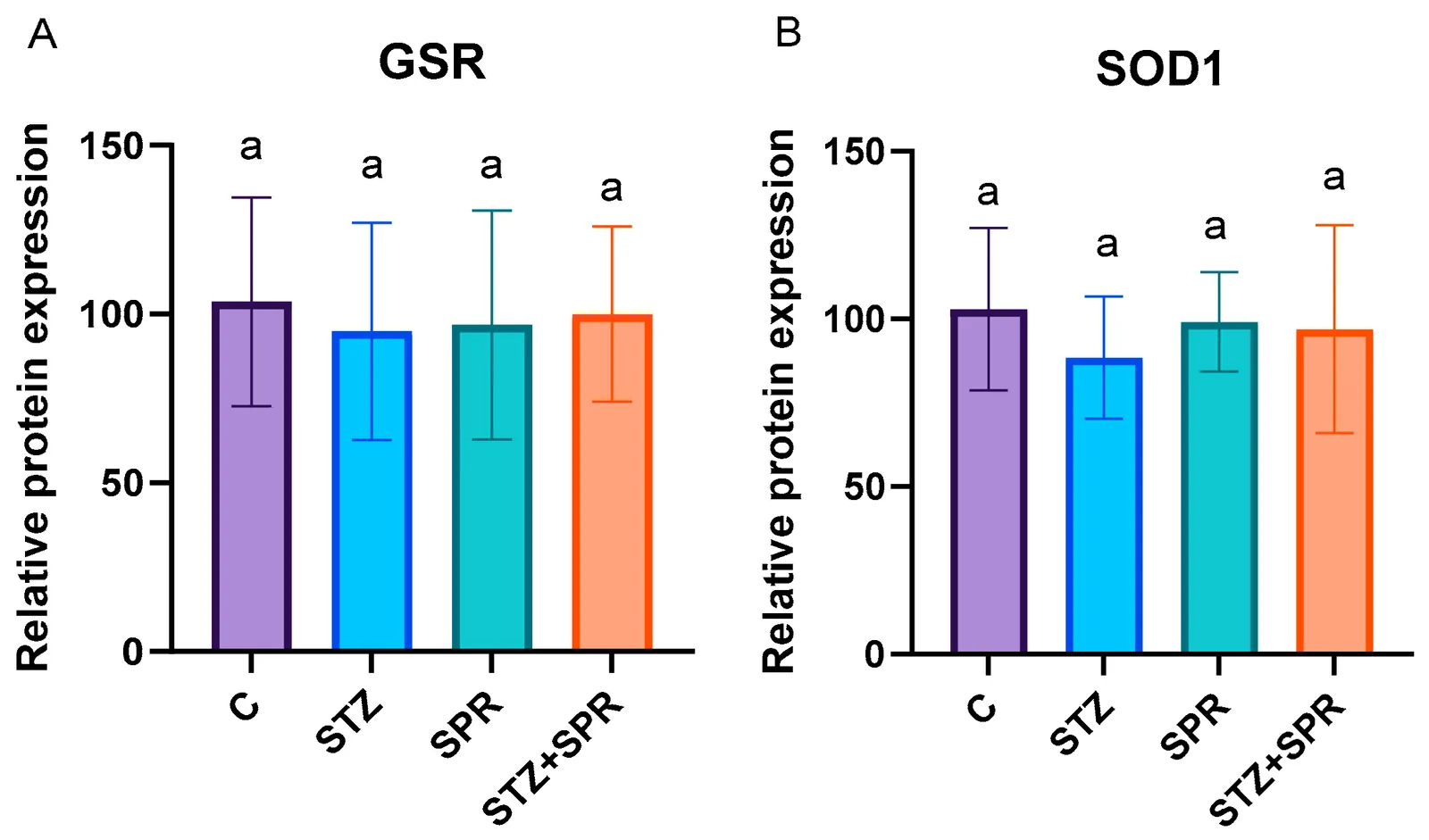
Effect of dietary supplementation with baked freeze-dried sprats on the hepatic protein expression of SOD1, GSR. Statistical analysis was performed using one-way ANOVA followed by Duncan’s post hoc test. Values marked with the same letter (a) are not differ significantly at *p* ≤ 0.05. Protein expression of GSR (**A**), SOD1 (**B**). Data are presented as mean ± SD (*n* = 6). Protein expression was normalized to β-actin and expressed relative to the control group (C = 100%).

The relative expression of proteins of glutathione reductase (GSR) and superoxide dismutase (SOD) in the liver was not affected by various dietary treatments (Figure 2).

## 3. Discussion

To the best knowledge of the authors of this manuscript, we present unique data regarding the effect of the addition of baked sprats to experimental diets on oxidative stress, immune response and thyroid hormones metabolism. Based on the current literature review, only a few articles have been published in which beneficial effects of sardine proteins or oil was evaluated, mainly on lipid metabolism [26,27,28]. In this study, we decided to use baking sprats because, based on our previous research, the baking process was one of the best in the case of the nutritional value [23] and organoleptic evaluation. The increase in dry matter is expected and results from moisture loss during baking. The changes in protein and fat proportions likely reflect changes in the macronutrient distribution during various thermal treatments, including baking. During the baking process, part of the lipid fraction may melt and drain from the tissue matrix, which reduces the fat share and increases the relative protein content on a dry matter basis. The decrease in ash content after baking may reflect losses of the mineral fraction associated with the release of tissue fluids. Our finding are similar to previously published data [23,29]. The proximate analysis of baked sprats was necessary to properly balance experimental diets for rodents, in which the macronutrient composition and the energy contribution of the added sprats were taken into account.

In our study, the lower body weight gain in the STZ + SPR group and the tendency of lower body weight gain in the STZ and SPR groups can be explained by the possibility of lower absorption of long-chain fatty acids from sprats, as these could form insoluble salts with Mg^2+^ or Ca^2+^ and therefore were not absorbed. Additionally streptozotocin selectively destroys β cells of the islets of Langerhans of the pancreas. In the absence of insulin, glucose cannot be transported into cells as a source of energy. This results in a greater reliance on fatty acids as an energy source, reducing the body’s ability to utilize protein and other nutrients, and ultimately leading to reduced weight gain [30,31]. At the same time, no significant differences were observed in the mass of the liver, kidneys, or heart among the experimental groups (Table 2). The observed changes in body mass may therefore have been functional or biochemical in nature, rather than being reflected in organ mass. High inter-individual variability may also have masked small differences. Loula et al. [27] reported that feeding rats a hypercholesterolemic diet with highly purified proteins from sardines did not affect weight gain. However, they found that the liver mass was significantly higher in the group of rodents fed with highly purified sardines proteins as compare to the control group. These results are opposite to our study. Additionally, Mir et al. [28] reported that the addition of sardine proteins to the hypercholesterolemic diet did not affect body weight gain and the liver weight as compared to the control group.

Diets enriched with sprats were better sources of the evaluated minerals, which could have affected their concentration in the urine, feces, liver and kidneys. We have found that after 5 weeks of feeding rats with experimental diets, the concentration of Ca was significantly higher in the feces of the SPR and STZ + SPR groups. In the case of Mg, in the 5th and 9th weeks of the experiment, its content in feces was significantly higher in the groups receiving sprats (SPR and STZ + SPR) compared with the groups without sprats addition (C and STZ). The increased supply of minerals together with the addition of sprats (minerals were not balanced in the diet) leads to an increase in their pool in the intestinal lumen. Part of this pool was excreted with feces due to limitations of absorption probably caused by the formation of poorly soluble complexes (e.g., calcium salts with phosphates, calcium or magnesium soaps with long-chain fatty acids) [31,32,33,34]. An increase in fecal excretion therefore does not have to mean a deterioration of mineral absorption, but may be an indicator of excess intake relative to absorption capacity [31,32,35,36,37]. We have also found that Mg and Ca excretion in urine did not show significant differences between groups, which indicates effective control of rodents’ organism at the level of renal filtration and reabsorption. Studies on rat models in the context of calcium homeostasis show that the kidneys maintain Ca concentration through filtration and reabsorption (including with the participation of the calcium-sensing receptor localized in the kidney and hormonal mechanisms), which stabilizes the loss of this mineral with urine [38,39]. The kidneys can stabilize the concentrations of these elements through the regulation of reabsorption; therefore, under conditions of moderate changes in intake, differences are more often observed in feces than in urine [38].

We have found that after 9 weeks of the experiment, Fe excretion in urine was significantly lower in the STZ + SPR group compared with its content in urine of the C and SPR groups. Albeit, Fe content significantly increased in the liver and kidneys of rats fed SPR and STZ + SPR diets (Table 3). This can be explained by good absorption of Fe from sprats [40]. Iron in the human body is not only necessary for hemoglobin synthesis but also for the synthesis of many enzymes, including these involved in the mitochondrial respiratory chain, peroxidases or catalases. It was reported that small fishes as the source of Fe^2+^ can improve iron status and also prevent cardiovascular diseases [41,42]. On the other hand iron is also important to reduce oxidative stress. Higher concentration of iron in the kidneys, especially in STZ + SPR rats, can be connected with higher production of antioxidant enzymes and protection from oxidative stress caused by impaired glucose metabolism in the organism of STZ rats (Table 3 and Table 4). This hypothesis can be confirmed by the content of Zn, Cu and Se in the kidney and the liver of rats, especially in the STZ + SPR group (Table 3), and the higher activity of GPx and SOD in the serum of rats from the SPR and STZ + SPR groups. Zn and Cu are essential micronutrients involved in the reduction of oxidative stress and in the immune response [20,43]. It is well known that Zn and Cu are the antagonistic trace elements, which are competitors in the intestinal lumen for the same transporter proteins and metallothioneins, which regulate the intracellular distribution, release, and transport of both minerals [43]. This may explain the higher content of Zn in the kidneys in the STZ and STZ + SPR rats compared to the Zn content in the kidneys in the C and SPR groups. It is also important that zinc and copper are necessary for synthesis of the SOD enzyme. We have found that SOD activity was significantly higher in the serum of rats in the STZ + SPR groups (Table 4). This positive change can be explained by the higher concentration of Zn and Cu in the liver and kidneys, which could increase the synthesis of SOD in STZ + SPR rats.

Higher concentration of selenium in the liver, kidney (Table 3) and serum in the form of the Se(IV) of rats fed diets with SPR addition (Table 4) could be explained by the good bioavailability of selenium from sprats and the synthesis of the GPX enzyme. Selenium also plays an important role in thyroid hormones metabolism because it is necessary for synthesis deiodinases (1–3) [44]. We have found that the T4 concertation was significantly higher in the SPR and STZ + SPR groups as compared to the T4 concentration in the serum of rats in the C group. This may be explained by the lower concentration of selenium in the form of Se_2_Cys, which is necessary for the synthesis of deiodinases, especially Dio1 and Dio2 which play important role in conversion of T4 to T3. In the thyroid gland the expression of the *Dio1* gene was relatively higher in the STZ + SPR group as compared to other experimental groups. Expression of *Dio2* and *Dio3* was significantly lower in the STZ + SPR thyroid glands compared to the C group. In the liver, the expression of Dio1 was significantly higher as compared to the STZ group (Table 4 and Table 5). This is an important finding of our study, and it can be explained also by the lower concentration of Se_2_Cys in the STZ + SPR group, which probably was used for the synthesis of SOD (Table 4 and Table 6). The other reason for the higher expression of Dio1 in the thyroid gland can be explained by the above mentioned higher concentration of T4 hormone in the SPR and STZ + SPR groups compared to the C group. However, the same concentration of T_3_ in all experimental groups can explain why *Dio2* expression was lower.

The concentration of TRIAC as the metabolite of T3 and T4 was significantly lower in the serum of the STZ + SPR group and tended to be lower in the serum of SPR and STZ rats. It also may be explained by the high concertation of T_4_ and the deficiency of Se_2_Cys to synthetize deiodinases.

We have also found that expression of the *Txnrd1* gene was significantly higher in the SPR and STZ + SPR thyroid glands compared to the expression of this gene in the thyroid gland of the C and STZ groups. The *Txnrd1* gene encodes the tioredoxin 1 enzyme, which is responsible for the inactivation of the excess content of H_2_O_2_ in the thyroid gland and protects thyrocytes from peroxidative damage. Similarly, the GPX1 and GPX3 enzymes are important in the redox status of the thyroid gland [31,43]. However, in our study, we have found that expression of the *Gpx1* gene was significantly lower in the thyroid gland of the STZ + SPR group. This could decrease synthesis of the *GPX 1* enzyme. This can be also explained by the fact that the rodents organism had a proper amount of the divalent iron. This caused a higher concentration of Fe in the liver and kidneys, which may result in the initiation of the Fenton reaction, in which free OH radicals are produced. On the other hand, oxidative stress caused by disturbed glucose metabolism could influence the increased synthesis of catalase, for example, which contains iron to reduce oxidative stress. It can be suggested that selenium, zinc, iron and copper as components of the antioxidant enzymes in the organism of rats with impaired glucose metabolism were important components to reduce oxidative stress, modulating iodine metabolism and immune responses.

Changes observed at the transcriptional level of protein were not consistently reflected in hepatic protein expression. In particular, alterations in hepatic *Sod1* gene expression were not accompanied by significant differences in SOD1 protein expression. This difference may result from post-transcriptional and translational regulation, differences in mRNA and protein turnover, and the temporal dynamics of these responses. At the same time, significant changes in serum antioxidant enzyme activities, including SOD, GR, and GPx, indicate that changes in the systemic antioxidant status may occur independently of hepatic protein expression. Therefore, transcriptional, protein, and enzymatic activity data should be interpreted as complementary but distinct levels of regulation.

Based on the results of the iodine content in rodents, it can be concluded that sprats are a readily available source of iodine. This can be confirmed by the effective T4 synthesis and high iodine concentration in the liver and kidneys. Conversely, low deiodinase activity could have resulted in reverse triiodothyronine (rT3) production. Furthermore, the TSH and T_3_ concentration remained unchanged compared to the group C, which suggests that T_3_ synthesis was sufficient in the other groups and that, therefore, excess T_4_ was metabolized. Wang et al. [45] showed that excessive iodine intake increased iodinated thyroid hormone precursors, particularly DIT, but decreased *Dio1* expression in the thyroid gland, and the T3/T4 ratio. What is more, we have found that the concentration of iodine in urine was affected by the experiment conditions (Table 3). In addition, the lower concentration of TRIAC in the STZ + SPR group suggests that under conditions of oxidative stress and the addition of sprats to the diet, the profile of thyroid hormone metabolites also may change [46]. It is possible that the pathways shift toward maintaining appropriate levels of active T3, with simultaneous accumulation of T4 (e.g., through modulation of deiodinases and/or hormone transport), which is reflected in the results of the expression of selected genes active in the thyroid [46,47,48].

Therefore, the significantly higher urinary iodine excretion observed in SPR-fed rats indicates that iodine supplied with sprats was well absorbed and entered the systemic iodine pool. Urinary iodine reflects both iodine availability and renal elimination, as more than 90% of ingested iodine is eliminated via the kidneys within 24–48 h [49,50]. Similar relationships have been reported in dietary studies in Wistar rats, where increased iodine intake was accompanied by significantly higher urinary iodine excretion and tissue iodine concentrations [51]. In the present study, significantly higher urinary iodine excretion was accompanied by significantly higher iodine concentrations in the liver and kidney, supporting the bioavailability and systemic distribution of iodine supplied with the SPR diet.

We have also found that salicylic acid, which has anti-inflammatory properties, was measured in a higher concentration in the serum of the C group, and its concentration decreased in the SPR group and tended to be lower in the serum of the STZ and STZ + SPR groups (Table 4). However, the available literature lacks data on salicylic acid concentration and its presence in the body of rats. Taking into account that salicylic acid is produced by plants, not by humans or animals, the likely source of salicylic acid was cornstarch. However, limited data demonstrate that intake of salicylic acid very fast reduces the T3 and T4 concentration in the human body [52]. That was not confirmed in this study. Increased T4 synthesis could have resulted from impaired energy homeostasis and the resulting increased metabolic demand. At the same time, T3 and TSH did not differ significantly between the presented groups.

## 4. Materials and Methods

### 4.1. Proximate Analysis of Sprats

Sprats (SPR) were obtained from domestic fisheries and purchased from a fish wholesaler in Koszalin (Poland). Prior to thermal processing, the fish were cleaned by removing the heads and fins. Baking was performed on fresh samples; the processing time was selected based on the literature data [23,53] and experimentally verified with consideration of consumer acceptance. The remaining material was freeze-dried using a laboratory freeze-dryer (Christ Alpha 1–4, Martin Christ Gefriertrocknungsanlagen, Osterode am Harz, Germany), and the resulting product was stored at −80 °C until future analyses and for experimental diets preparation. To properly balance the experimental diets, basic chemical composition was performed. The concentration of proteins and crude fat were taken into account to properly balance experimental diets. Total protein (AOAC method 950.36), crude fat (AOAC method 935.38), and ash (AOAC method 930.05) were analyzed according to AOAC procedures [54].

### 4.2. Animal Study

Five-week-old male Wistar rats (n = 48) with a mean body weight of 90–125 g were obtained from a certified breeding facility included in the Polish governmental list based on the Journal of Laws [55]. Experimental procedures were conducted in accordance with Polish ethical standards and were approved by the 1st Local Ethical Committee for Animal Experiments in Kraków (Resolution No.585/2021). Animals were acclimated for 10 days on a standard laboratory diet. During the experiment, rodents were kept in plastic or metabolic cages under a controlled temperature (25 °C) and a 12/12 h light/dark cycle. After acclimatization, rats were randomly allocated into four experimental groups (n = 12). On day 8, following a 7-day acclimation period, streptozotocin (STZ; Sigma-Aldrich, cat no. S0130; Saint Louis, MO, USA) was administered intraperitoneally (65 mg/kg body weight, dissolved in citrate buffer (pH 4.5)) to 24 animals. Streptozotocin was used to induce impaired glucose metabolism and oxidative stress. After injection, animals were continuously monitored to assess behavior and general physical condition. Experimental diets were prepared based on the AIN-93G diet [56], with an addition of baked freeze-dried sprats (Table 6). Rats from groups C and SPR were put into metabolic cages for 5 days to collect samples of urine and feces to assess the biological value of proteins from baked sprats (Table 6). During this period, experimental diets delivered 10% of protein (Table 6). Diet intake and uneaten diet were monitored. Also, rats from groups STZ and STZ + SPR were fed a similar diet. For the rest of the experimental period, the diet was balanced to exchange soybean oil with fat from fish, e.g., 188.37 g/kg of fish delivered 70 g of fat and this amount was added to the experimental diet. Casein content was decreased in the diet for the SPR and STZ + SPR group to 87.3 g to balance protein content. Group I-control (C) and group II (STZ) were fed the AIN-93G diet. In weeks 5 and 9, all animals were moved to metabolic cages for 5 days for collection of urine and feces for the assessment of selected minerals content. During weeks without urine and feces samples collection, rodents were housed in plastic cages (two animals per cage). Feed and water were provided *ad libitum*. Body weight gain was recorded weekly throughout the 10-week experimental period. At the end of the experiment (10 weeks), animals were euthanized after a 12 h fasting period under inhalation anesthesia (isoflurane 4%, Baxter, Warszawa, Poland). Prior to the procedure, the animals received butorphanol (Morphasol; LIVISTO-aniMedica GmbH, Senden-Bösensell, Germany) for analgesia. Blood was collected by cardiac puncture into tubes without anticoagulant. After centrifugation (4000× *g*, 10 min), the plasma or the serum was obtained and stored at −80 °C until further analyses. The liver, kidneys and heart were dissected, washed in cold 0.9% NaCl, dried with laboratory tissue paper, weighed, and stored at −80 °C until analysis.

### 4.3. Apparent Protein Quality Indices

Apparent protein digestibility (TD), apparent biological value (BV), and apparent net protein utilization (NPU) were determined using the nitrogen balance method. During the 5-day balance period, feed intake, feces, and urine were quantitatively collected from individually housed rats maintained in metabolic cages. Nitrogen intake was calculated from dietary protein intake, determined by the Kjeldahl method (AOAC Official Method 950.36), using a nitrogen-to-protein conversion factor of 6.25. Fecal and urinary nitrogen contents were also determined by the Kjeldahl method. Apparent TD, apparent BV, and apparent NPU were calculated using the following equations:

$$TD(\%)=\frac{\left(Ni-Nf\right)}{Ni}\times 100$$

$$BV(\%)=\frac{\left(Ni-Nf-Nu\right)}{\left(Ni-Nf\right)}\times 100$$

$$NPU(\%)=\frac{\left(Ni-Nf-Nu\right)}{Ni}\times 100$$

where Ni—nitrogen intake, Nf—fecal nitrogen excretion, and Nu—urinary nitrogen excretion. Calculations were performed according to the principles of the nitrogen balance method [57,58].

### 4.4. Determination of Minerals in Animal Tissues, Urine, and Feces

Prior to analysis, urine samples were stored in a freezer at −20 °C. Feces and organs (kidneys, liver) were freeze-dried. After drying, organs were weighed and homogenized in a mortar and pestle. The prepared material was used for subsequent determination of I, Mg, Ca, Fe, Zn, Se and Cu. The elements were determined by inductively coupled plasma tandem mass spectrometry (ICP-MS/MS) using a triple-quadrupole instrument (iCAP TQ ICP-MS; Thermo Fisher Scientific, Bremen, Germany).

The total iodine concentration in feces, liver and kidneys was determined after alkaline extraction with tetramethylammonium hydroxide (TMAH). The analysis by ICP-MS/MS was performed according to the procedure described by Smoleń et al. [59] and the Polish–European Standard PN-EN 15111:2008 [60]. For iodine determination, urine samples were thawed and mixed, then 4.8 mL of each was placed in a polypropylene tube. Next, 0.2 mL of tetramethylammonium hydroxide (TMAH) was added. The mixture was incubated, mixed, and diluted 200-fold with triple-distilled water, and next, the iodine content was determined using the previously mentioned ICP-MS/MS technique.

Iodine in urea, feces and organs (kidneys, liver) was quantified by ICP-MS/MS using tellurium as an internal standard. Calibration was based on a five point iodine standard curve in the range 1–200 µg I/L, L for feces, kidneys, and liver (or six-point standard curve in the range 1–4000 µg I/L for urine), prepared in the same TNAH matrix as the analyzed samples.

Concentrations of Mg, Ca, Fe, Zn, Se and Cu in urea, feces, kidneys, and liver samples were determined after microwave digestion. Prior to measurement, samples were mineralized as follows [61]: 0.5 g of dried material (feces, liver and kidney) or 5 mL urea was placed in 55 mL TMF vessels and digested in 10 mL of 65% Suprapur HNO_3_ (Merck, No. 100443.2500) using a Mars 5 Xpress microwave digestion system (CEM, Matthews, NC, USA). The digestion program comprised a ramp to 200 °C (15 min), followed by a hold at 200 °C (20 min). After cooling, digests were quantitatively transferred into 25 mL volumetric flasks and made up to volume with double-distilled water. Finally, mineral concentrations were measured using a mentioned ICP-MS/MS spectrometer. Depending on the concentrations of Mg, Ca, Fe, Zn, Se, and Cu in the samples, multi-point calibration curves were used. The standard concentrations were appropriately matched to the concentrations of these elements in the urea, feces, kidneys, and liver samples.

The quality and accuracy of the measurements of the individual elements by the ICP-MS/MS technique were verified by means of: measurement of quality control (QC) samples and measurements of elements concentrations in reference material (CRM). The following CRM materials were used: garlic No. NCS ZC73020 (NCS Testing Technology Co., Ltd., Gaoliangqiaoxie Street, Haidian District, Beijing, China) and RECIPE^®^ ClinChek^®^ Urine Control, lyophilized, for Trace Elements, Level I No. REC-8849 (RECIPE—CHEMICALS + INSTRUMENTS GmbH, Munich, Germany). In CRM Garlic NCS ZC73020 samples, the average recoveries for iodine, Se, Cu, Zn, Ca, Mg and Fe, were: 89.5%, 100.7%, 90.3%, 111.7%, 98.2%, 103.2% and 87.4%, respectively. In urine CRM No. REC-8849 samples, the average recoveries for iodine, Se, Cu, Zn, Mg and Fe, were: 98.1%, 92.7%, 128.0%, 96.9%, 107.3% and 114.8% respectively; content of Ca was not certified in this CRM urine material. The average recoveries in QC samples for iodine, Se, Cu, Zn, Ca, Mg and Fe were 101.3%, 102.8%, 103.8%, 100.6%, 106.1%, 96.6% and 100.6%, respectively.

### 4.5. Selected Biochemical Parameters Analyses in the Serum

Serum was analyzed to determine glutathione reductase (GR) activity using the Glutathione Reductase Assay Kit (Cat. No. KX03401, BQC Redox Technologies, Oviedo, Asturias, Spain), glutathione peroxidase (GPx) activity using the Glutathione Peroxidase (GSH-PX) Assay Kit (Cat. No. BC0121, ELK Biotechnology, Wuhan, China), and superoxide dismutase (SOD) activity using the SOD Activity Assay Kit (Cat. No. KB03011-100, BQC Redox Technologies, Spain). Thyroid-stimulating hormone (TSH) was measured using a Rat Thyroid Stimulating Hormone ELISA kit (Cat. No. AR E-8600R, Labor Diagnostika Nord GmbH & Co. KG, Nordhorn, Germany). Triiodothyronine (T3) concentration was determined using the Mouse/Rat Triiodothyronine (T3) ELISA kit (Cat. No. T3043T-100, Calbiotech, El Cajon, CA, USA). Thyroxine (T4), iodotyrosine, triiodothyroacetic acid (TRIAC), tetraiodothyroacetic acid (TETRAC) and salicylic acid (SA) were quantified by liquid chromatography–tandem mass spectrometry (LC–MS/MS) using an Ultimate 3000 system (Thermo Scientific Waltham, MA, USA) coupled to a QTRAP 4500 mass spectrometer (Sciex, Marlborough, MA, USA). Chromatographic separation was performed on a Luna Phenyl-Hexyl column (3 µm, 100 Å; 150 mm × 3 mm; Phenomenex, Torrance, CA USA) using a gradient of mobile phases: A, water containing 0.3% formic acid; B, methanol containing 0.3% formic acid. The total run time was 15 min, and the injection volume was 10 µL. Detection was carried out in multiple reaction monitoring (MRM) mode with electrospray ionization (ESI) in negative-ion mode, monitoring the iodotyrosine (transition *m*/*z* 306.1 → 126.8), TRIAC (transition *m*/*z* 620.8 → 576.6), TETRAC (transition *m*/*z* 746.6 → 126.7) and SA (transition *m*/*z* 136.8→93.1), as well as in positive-ion mode, monitoring the T4 (transition *m*/*z* 777.7 → 731.8). Data acquisition and processing were performed using Analyst version 1.7 software (Sciex, Marlborough, MA, USA).

Serum concentrations of selected cytokines were determined using the Bio-Plex Pro Rat Cytokine Group I 7-Plex Assay (Cat. No. 12023390, Bio-Rad Laboratories, Hercules, CA, USA), according to the manufacturer’s instructions. The assay simultaneously quantified interferon-γ (IFN-γ); interleukin (IL)-1α, IL-1β, IL-2, IL-6, IL-10; and tumor necrosis factor-α (TNF-α). Fluorescence intensity was measured using the Bio-Plex 200 System (Bio-Rad Laboratories, Hercules, CA, USA), operated with high photomultiplier tube (PMT) settings. Cytokine concentrations were calculated from five-parameter logistic (5-PL) standard curves using Bio-Plex Manager™ software, version 6.2 (Bio-Rad Laboratories, Hercules, CA, USA) and expressed as pg/mL. Samples with concentrations below the assay detection limit were classified as out of range (OOR), according to the manufacturer’s criteria, and were excluded from statistical analyses.

### 4.6. Expression of Selected Genes and Proteins

#### 4.6.1. RNA Isolation and cDNA Synthesis

Total RNA was isolated from 15 to 20 mg of frozen rat tissues (thyroid gland, liver, lungs, heart and kidney) using the Total RNA Mini Plus kit (Cat. No. 036-25; A&A Biotechnology, Gdynia, Poland), according to the manufacturer’s instructions. RNA concentration and purity were assessed using a Multiskan Go spectrophotometer (Thermo Fisher Scientific, Waltham, MA, USA) by measuring absorbance at 260 and 280 nm. All samples exhibited A260/A280 ≥ 1.7 and A260/A230 ≥ 1.9, confirming suitability for downstream molecular analyses. All RNA samples were handled under RNase-free conditions and stored appropriately prior to downstream analyses. The same amount of RNA was used for cDNA synthesis for each sample, and the RT-qPCR analyses were performed under identical experimental conditions. The isolated RNA (mRNA) was reverse-transcribed using the iScript RT Supermix for RT-qPCR kit (Cat. No. 170-8840; Bio-Rad, Hercules, CA, USA).

#### 4.6.2. Gene Expression Analysis by Real-Time Quantitative PCR (RT-qPCR)

The resulting cDNA was used for real-time PCR reactions performed in a mixture containing either TaqMan Fast Advanced Master Mix (Thermo Fisher Scientific; Cat. No. 4444556, Waltham, MA, USA) or SsoAdvanced Universal SYBR Green Supermix (Cat. No. 1725270, Bio-Rad Laboratories, Hercules, CA, USA).

Candidate genes were selected based on a comprehensive literature review and bioinformatic analysis using the UniProt Knowledgebase (UniProtKB), the National Center for Biotechnology Information (NCBI Gene), Gene Ontology (GO), and the Kyoto Encyclopedia of Genes and Genomes (KEGG) for Rattus norvegicus. Genes were chosen according to their documented expression in the thyroid, liver, kidneys, heart and lungs, as well as their established roles in antioxidant defense, cellular redox homeostasis, and thyroid hormone metabolism. Accordingly, the expression of Sod1, Gpx1, Gpx3, Txnrd1, Txnrd2, Dio1, Dio2, and Dio3 was evaluated.

Gene expression was analyzed using appropriate primers and probes. TaqMan assays were used for *Gpx1* (Assay ID: Rn00577994_g1), *Gpx3* (Rn00574703_m1), *Sod1* (Assay ID: Rn00566938_m1), *Txnrd1* (Rn01503798_m1) and Txnrd2 (Rn00574868_m1), whereas *Dio1*, *Dio2*, and *Dio3* were amplified using primers synthesized by Genomed S.A. (Warsaw, Poland). Primers were ordered in the 0.02 µmol synthesis scale with standard purification (ethanol precipitation), according to the manufacturer’s offer.

The primer sequences used for SYBR Green-based analyses were as follows: Dio1 forward 5′-CTGGTTCGTCCTGAAGGTCC-3′ and reverse 5′-GGTTTACCCTGTGGCGTGAG-3′; Dio2 forward 5′-AAAAATTGGCCGCTCCACAC-3′ and reverse 5′-TGCCCGGATGACTTCCTCTA-3′; and Dio3 forward 5′-CATCTGCGTATCCGACGACA-3′ and reverse 5′-AAAATTGAGCACCAACGGGC-3′. Cycling conditions were adjusted to the assay chemistry. For TaqMan Fast Advanced Master Mix, reactions started with an initial denaturation at 95 °C for 20 s, followed by 40 cycles of denaturation at 95 °C for 1 s and annealing/extension at 60 °C for 20 s. For reactions performed using SsoAdvanced Universal SYBR Green Supermix (BioRad, Hercules, CA, USA), initial denaturation was carried out at 95 °C for 2–3 min, followed by 40 cycles of denaturation at 95 °C for 5–10 s and annealing/extension at 60 °C for 30 s. RT-qPCR was performed using the CFX96 Touch™ Deep Well Real-Time PCR Detection System (Bio-Rad, Hercules, CA, USA). Each of the four experimental groups comprised biological material collected from eight animals, and three technical replicates were performed for each organ sample. Expression levels were analyzed using relative quantification based on Ct values by comparing experimental samples with controls and correcting for amplification efficiency. Expression was normalized to the reference genes *Rn45s* for TaqMan^®^ assays and *Gapdh* and Rn45s for SYBR Green-based analyses.

#### 4.6.3. Protein Expression Analysis by Western Blot

The target proteins were selected based on an extensive literature review and bioinformatic analysis using the UniProt Knowledgebase [62], the National Center for Biotechnology Information [63], and Gene Ontology [64] annotations for Rattus norvegicus. Candidate proteins were selected according to their documented expression in rat liver and their established roles in antioxidant defense, reactive oxygen species detoxification, and the maintenance of cellular redox homeostasis.

Frozen liver tissue samples (approximately 30 mg) were homogenized on ice in an IGEPAL-based lysis buffer (50 mM Tris-HCl (Cat. No. TRS002.500, BioShop Canada Inc., Burlington, ON, Canada), pH 8.0, 50 mM NaCl (Eurochem BGD Sp. z o.o., Tarnów, Poland), 5 mM EDTA (Cat. No. 118798103.1000, Chempur, Piekary Śląskie, Poland), and 0.5% IGEPAL CA-630 (Cat. No. I3021-50ML, Merck, Darmstadt, Germany), supplemented immediately before use with a Protease Inhibitor Cocktail (100×; Cat. No. 5871S, Cell Signaling Technology, Danvers, MA, USA), phenylmethylsulfonyl fluoride (PMSF; Cat. No. 8553S, Cell Signaling Technology, Danvers, MA, USA) to a final concentration of 1 mM, and Benzonase^®^ Nuclease (Cat. No. E1014-5KU, Merck, Darmstadt, Germany) in the presence of MgCl_2_ (Cat. No. 363-116120500-100G, Chempur, Piekary Śląskie, Poland). Homogenates were centrifuged at 20,000× *g* for 20 min at 4 °C, and the resulting supernatants were collected. Protein concentration was determined using the Pierce™ BCA Protein Assay Kit (Cat. No. 23225, Thermo Fisher Scientific, Waltham, MA, USA). Equal amounts of protein (30 µg per lane) were mixed with 4× Laemmli sample buffer (Cat. No. 1610747, Bio-Rad Laboratories, Hercules, CA, USA) containing β-mercaptoethanol (Cat. No. M6250, Merck (Sigma-Aldrich), St. Louis, MO, USA) and denatured at 95 °C for 5 min. Proteins were separated by SDS-PAGE using 4–15% Mini-PROTEAN^®^ TGX™ Precast Protein Gels (Cat. No. 4561083, Bio-Rad Laboratories, Hercules, CA, USA) together with the Perfect Rainbow Protein Ladder (Cat. No. E3212-01, EURx, Gdańsk, Poland). Following electrophoresis, proteins were transferred onto 0.2 µm nitrocellulose membranes using Trans-Blot^®^ Turbo™ Mini Nitrocellulose Transfer Packs (Cat. No. 1704158) and the Trans-Blot^®^ Turbo™ Transfer System (Cat. No. 1704150, Bio-Rad Laboratories, Hercules, CA, USA).

Following transfer, membranes were blocked for 1 h at room temperature in either 5% non-fat dry milk or 5% bovine serum albumin (BSA; Cat. No. A9647, Sigma-Aldrich, St. Louis, MO, USA), prepared in 1× Tris-buffered saline containing 0.1% Tween^®^ 20 (TBST), prepared from 10× Tris Buffered Saline (Cat. No. 1706435) and Tween^®^ 20 (Cat. No. 1706531, Bio-Rad Laboratories, Hercules, CA, USA), depending on the primary antibody. Membranes were incubated overnight at 4 °C with primary antibodies diluted 1:1000 in the corresponding blocking buffer. The following primary antibodies were used: β-actin (Cat. No. 4970L, Cell Signaling Technology, Danvers, MA, USA), HO-1 (Cat. No. 43966S, Cell Signaling Technology, Danvers, MA, USA), SOD1 (Cat. No. 37385S, Cell Signaling Technology, Danvers, MA, USA) and glutathione reductase (GSR) (Cat. No. STJA0006922-100, St John’s Laboratory Ltd., London, UK). After three washes in TBST (5 min each), membranes were incubated for 1 h at room temperature with an HRP-conjugated anti-rabbit IgG secondary antibody (Cat. No. 7074S, Cell Signaling Technology, Danvers, MA, USA) diluted 1:1000 in the appropriate blocking buffer. Immunoreactive bands were visualized using Clarity™ Western ECL Substrate (Cat. No. 1705060, Bio-Rad Laboratories, Hercules, CA, USA) and detected with the ChemiDoc™ MP Imaging System (Bio-Rad Laboratories, Hercules, CA, USA). Where appropriate, membranes were stripped using Restore™ PLUS Western Blot Stripping Buffer (Cat. No. 46430, Thermo Fisher Scientific, Waltham, MA, USA), re-blocked, and reprobed with another primary antibody. Band intensities were quantified using Image Lab™ Software Version 6.1.0 (Bio-Rad Laboratories, Hercules, CA, USA). The intensity of each target protein was normalized to β-actin, and the results were expressed as the target protein/β-actin ratio.

### 4.7. Statistical Analysis

Data are presented as mean ± standard deviation (SD). When assumptions for parametric analysis were met, one-way analysis of variance (ANOVA) was applied at a significance level of *p* ≤ 0.05, and between-group differences were evaluated using Duncan’s post hoc test. When data did not meet normality assumptions, the non-parametric Kruskal–Wallis test was used, followed by Dunn’s post hoc test with Bonferroni correction. Statistical significance in figures is indicated by different letters at *p* ≤ 0.05. In addition, effect sizes for pairwise comparisons were calculated as Hedges’ g using Microsoft Excel 2019 (Microsoft Corporation, Redmond, WA, USA), based on the difference between group means standardized by the pooled standard deviation and corrected for small-sample bias. Effect sizes were interpreted as negligible (|g| < 0.2), small (0.2 ≤ |g| < 0.5), moderate (0.5 ≤ |g| < 0.8), and large (|g| ≥ 0.8). Statistical analyses were performed using Statistica version 13.1 PL (TIBCO Software Inc., Palo Alto, CA, USA).

## 5. Conclusions

The main novelty of this study is that selenium, zinc, iron and copper, as components of antioxidant enzymes in the organism of rats with impaired glucose metabolism, were important components, modulating iodine metabolism to reduce oxidative stress. Despite the lack of changes in the concentration of cytokines, which are important markers of inflammation, differences in the activity of antioxidant enzymes, e.g., GPX and SOD, suggest that oxidative stress was reduced, which could reduce the risk of inflammation developing. The differences found may indicate that sprats, which are a source of many nutrients and interfering components, could contribute to improving immune system function; however, more studies are needed.

## Figures and Tables

**Table 1:** Nitrogen balance and protein utilization indices in rats fed the control and sprat diets.

Parameter	C	SPR	*p*-Value	Hedges’ *g*
Body weight gain *	3.67 ± 3.39	9.83 ± 2.76	<0.001	1.453
Feed intake *	56.87 ± 1.79 b	67.62 ± 3.15 a	<0.001	4.053
Protein intake *	5.69 ± 0.18 b	6.76 ± 0.31 a	<0.001	4.049
Nitrogen intake [mg]	909.90 ± 28.63 b	1081.87 ± 50.36 a	<0.001	4.053
Dry fecal mass *	2.74 ± 0.22 a	2.75 ± 0.58 a	0.919	0.040
Fecal nitrogen content [%]	0.96 ± 0.05 b	1.46 ± 0.32 a	<0.001	2.103
Fecal nitrogen excretion [mg]	26.18 ± 2.85 b	39.83 ± 12.52 a	0.001	1.451
Urinary nitrogen excretion [mg]	100.85 ± 14.25 a	115.37 ± 24.04 a	0.086	0.710
Nitrogen retention [mg]	782.88 ± 32.97 b	926.67 ± 56.10 a	<0.001	3.017
Apparent TD [%]	97.12 ± 0.30 a	96.32 ± 1.12 b	0.025	−0.950
Apparent BV [%]	88.57 ± 1.71 a	88.90 ± 2.39 a	0.697	0.155
Apparent NPU [%]	86.02 ± 1.71 a	85.62 ± 2.05 a	0.604	−0.208

* Presented in grams; parameter for whole period of collection of biological samples. Values are presented as mean ± SD. Values within a row marked with different letters (a, b) differ significantly at *p* ≤ 0.05. C—control group; SPR—rats fed diet with sprats; TD—apparent digestibility; BV—biological value; NPU—net protein utilization.

**Table 2:** Body weight gain and liver, heart and kidneys weights in experimental rats [g].

Parameters	C	STZ	SPR	STZ + SPR
Body weight gain	287.67 ± 33.92 a	249.00 ± 51.13 ab	257.83 ± 39.10 ab	210.00 ± 61.45 b
Liver	13.84 ± 2.36 a	14.39 ± 1.87 a	13.29 ± 2.26 a	14.36 ± 2.62 a
Kidneys	2.6 ± 0.36 a	2.6 ± 0.5 a	2.47 ± 0.37 a	3.07 ± 0.49 a
Heart	1.15 ± 0.08 a	1.22 ± 0.12 a	1.12 ± 0.15 a	1.12 ± 0.20 a

Values within a row marked with different letters (a,b) differ significantly at *p* ≤ 0.05. C—control group; STZ—streptozotocin-injected rats; SPR—rats fed diet with sprats; STZ + SPR—rats injected with streptozotocin and fed diet with sprats.

**Table 3:** Selected minerals content in urine, feces and organs of rats.

	C	STZ	SPR	STZ + SPR
Urine [ug/dm^3^]				
Week 5				
Mg	318,830 ± 71,120 a	318,930 ± 82,780 a	338,410 ± 95,540 a	227,050 ± 67,800 a
Ca	276,370 ± 113,850 a	180,890 ± 112,270 a	195,500 ± 62,030 a	179,950 ± 64,280 a
Fe	1487 ± 588 a	1787 ± 1105 a	1453 ± 488 a	770 ± 301 a
I	499.86 ± 121.15 b	504.40 ± 180.80 b	1208.08 ± 437.70 a	483.48 ± 150.48 b
Zn	845.35 ± 236.08 a	518.55 ± 159.18 a	631.18 ± 233.64 a	470.49 ± 206.50 a
Se	286.56 ± 111.52 b	341.38 ± 108.47 b	575.90 ± 188.23 a	246.64 ± 84.17 b
Cu	222.57 ± 42.12 b	242.90 ± 89.59 b	427.53 ± 92.37 a	256.77 ± 94.65 b
Week 9				
Mg	208,500 ± 125,320 a	239,170 ± 88,520 a	235,080 ± 68,570 a	192,350 ± 71,630 a
Ca	48,970 ± 21,030 a	56,350 ± 21,740 a	50,690 ± 21,370 a	49,080 ± 19,340 a
Fe	561.12 ± 183.12 a	278.25 ± 154.91 ab	400.71 ± 237.65 ab	117.57 ± 89.48 b
I	433.85 ± 184.58 ab	397.35 ± 193.89 ab	655.36 ± 190.02 a	263.44 ± 87.54 b
Zn	567.99 ± 129.83 a	343.96 ± 94.70 ab	321.97 ± 92.60 b	278.55 ± 167.98 b
Se	330.11 ± 92.14 ab	420.78 ± 173.76 a	367.29 ± 110.50 ab	156.14 ± 81.07 b
Cu	249.85 ± 80.43 ab	233.16 ± 59.83 ab	308.89 ± 75.36 a	157.01 ± 82.87 b
Feces [mg/kg s.m.]				
Week 5				
Mg	2887 ± 485 b	2865 ± 428 b	3978 ± 1668 a	5130 ± 499 a
Ca	21,289 ± 4407 b	22,959 ± 5703 b	25,847 ± 7947 a	35,502 ± 4439 a
Fe	596.01 ± 66.75 a	647.35 ± 86.75 a	597.90 ± 132.77 a	664.86 ± 80.12 a
I	0.743 ± 0.302 a	0.800 ± 0.411 a	1.478 ± 0.612 a	1.192 ± 0.210 a
Zn	581.07 ± 69.80 a	582.53 ± 97.72 a	532.32 ± 101.82 a	606.27 ± 92.95 a
Se	0.95 ± 0.23 a	0.99 ± 0.22 a	1.23 ± 0.42 a	1.37 ± 0.15 a
Cu	92.50 ± 16.02 a	91.08 ± 18.78 a	70.65 ± 6.59 a	79.30 ± 14.08 a
Week 9				
Mg	2762 ± 381 b	2832 ± 356 b	4575 ± 721 a	5285 ± 560 a
Ca	30,905 ± 4455 a	32,164 ± 7038 a	35,634 ± 3590 a	40,364 ± 5923 a
Fe	660.51 ± 85.82 a	712.30 ± 62.67 a	690.49 ± 54.68 a	687.24 ± 60.06 a
I	0.991 ± 0.542 a	0.928 ± 0.542 a	1.478 ± 0.413 a	1.328 ± 0.277 a
Zn	555.30 ± 51.90 a	575.05 ± 63.82 a	563.86 ± 106.39 a	603.75 ± 102.79 a
Se	1.23 ± 0.46 a	1.29 ± 0.53 a	1.51 ± 0.32 a	1.44 ± 0.29 a
Cu	104.47 ± 13.58 a	112.28 ± 17.24 a	97.33 ± 22.31 a	109.27 ± 15.60 a
Tissues [mg/kg d.m.]				
Liver				
Mg	674.68 ± 83.49 b	658.90 ± 58.15 b	750.77 ± 45.20 a	777.32 ± 53.41 a
Ca	117.94 ± 25.90 a	108.30 ± 28.70 b	134.27 ± 39.96 a	153.59 ± 32.73 a
Fe	286.62 ± 82.36 a	243.28 ± 60.17 b	351.66 ± 62.03 a	349.16 ± 71.35 a
I	80.24 ± 25.90 b	55.52 ± 12.15 b	732.59 ± 87.33 a	687.74 ± 210.79 a
Zn	82.33 ± 11.61 c	80.54 ± 7.61 c	107.76 ± 12.36 b	126.80 ± 20.70 a
Se	3.10 ± 0.39 b	2.95 ± 0.38 b	3.67 ± 0.45 a	3.52 ± 0.33 a
Cu	10.93 ± 1.44 b	10.87 ± 1.13 b	11.78 ± 1.28 b	14.38 ± 2.73 a
Kidney				
Mg	1321 ± 146 a	1368 ± 114 a	1279 ± 107 a	1380 ± 105 a
Ca	529.11 ± 199.10 a	468.55 ± 134.24 a	441.74 ± 128.34 a	377.97 ± 55.18 a
Fe	224.12 ± 18.08 b	237.58 ± 30.48 b	298.03 ± 38.01 a	318.65 ± 52.50 a
I	0.21 ± 0.06 b	0.18 ± 0.05 b	0.36 ± 0.03 a	0.33 ± 0.04 a
Zn	91.81 ± 10.20 b	101.82 ± 9.47 a	97.22 ± 8.85 b	116.53 ± 17.15 a
Se	4.75 ± 0.41 c	5.47 ± 0.91 c	6.70 ± 0.73 b	7.80 ± 0.68 a
Cu	43.84 ± 6.31 a	50.17 ± 18.71 a	27.47 ± 4.65 b	51.08 ± 21.43 a

Values within a row marked with different letters (a–c) differ significantly at *p* ≤ 0.05. C—control group; STZ—streptozotocin-injected rats; SPR—rats fed diet with sprats; STZ + SPR—rats injected with streptozotocin and fed diet with sprats.

**Table 4:** Selected biochemical parameters in the serum of rats fed experimental diets.

Treatment	C	STZ	SPR	STZ + SPR
TSH [ng/mL]	2.90 ± 0.11 a	2.82 ± 0.17 a	2.85 ± 0.14 a	2.75 ± 0.21 a
Iodotyrosine [ug/L]	1.41 ± 0.43 a	1.50 ± 0.27 a	1.41 ± 0.39 a	1.40 ± 0.29 a
T4 [ug/L]	6.98 ± 2.09 c	13.56 ± 2.49 ab	12.36 ± 4.48 b	16.33 ± 5.39 a
T3 [ng/mL]	0.87 ± 0.16 a	0.91 ± 0.20 a	0.95 ± 0.21 a	0.91 ± 0.16 a
Triiodothyroacetic acid [ug/L]	0.75 ± 0.70 a	0.45 ± 0.33 ab	0.47 ± 0.24 ab	0.43 ± 0.18 b
Tetraiodothyroacetic acid [ug/L]	0.45 ± 0.27 ab	0.65 ± 0.33 a	0.22 ± 0.15 b	0.52 ± 0.49 ab
Salicylic acid [ug/L]	15.77 ± 6.66 a	11.09 ± 4.75 ab	7.70 ± 2.13 b	11.02 ± 7.28 ab
SeUrea [ug/L]	13.07 ± 3.75 b	17.82 ± 4.67 a	14.79 ± 1.98 ab	15.20 ± 2.17 ab
SeMetCys [ug/L]	0.63 ± 0.43 a	0.84 ± 0.73 a	0.76 ± 0.46 a	0.34 ± 0.22 a
SeMetionin [ug/L]	0.42 ± 0.29 ab	0.33 ± 0.29 ab	0.34 ± 0.25 b	0.58 ± 0.23 a
Se_2_Cys [ug/L]	44.25 ± 6.91 a	38.87 ± 5.30 ab	32.72 ± 11.69 b	30.18 ± 10.40 b
Se(IV) [ug/L]	11.42 ± 2.19 b	12.82 ± 4.30 ab	14.38 ± 4.42 a	11.67 ± 3.05 ab
Se(VI) [ug/L]	0.42 ± 0.51 a	0.69 ± 0.93 a	0.82 ± 0.57 a	0.66 ± 0.29 a
I^−^ [ug/L]	13.68 ± 7.58 a	17.31 ± 6.89 a	17.01 ± 4.77 a	15.75 ± 2.96 a
GR [U/L]	51.40 ± 12.03 a	44.15 ± 9.64 ab	39.49 ± 9.35 b	34.05 ± 8.80 c
GPx [U/mL]	81.84 ± 1.79 c	89.67 ± 23.69 bc	99.60 ± 7.03 ab	108.07 ± 13.02 a
SOD [U/mL]	45.91 ± 7.80 b	40.44 ± 2.53 b	53.58 ± 18.43 ab	79.82 ± 2.17 a
Selected cytokines [pg/mL]				
IFN-γ	162.38 ± 82.31 a	197.12 ± 114.09 a	171.86 ± 77.71 a	147.48 ± 52.89 a
IL-1α	78.79 ± 38.05 a	81.91 ± 27.57 a	76.23 ± 23.94 a	76.13 ± 35.16 a
IL-1β	80.37 ± 71.85 a	55.17 ± 32.17 a	104.38 ± 89.44 a	67.39 ± 30.96 a
IL-2	2487 ± 758 a	2711 ± 1094 a	3016 ± 984 a	2363 ± 813 a
IL-6	196.20 ± 107.56 a	285.11 ± 153.79 a	218.75 ± 85.06 a	162.97 ± 68.79 a
IL-10	109.88 ± 59.34 a	137.16 ± 70.28 a	101.52 ± 52.07 a	118.24 ± 49.21 a
TNF-α	248.20 ± 117.29 a	259.11 ± 156.65 a	290.91 ± 227.73 a	225.75 ± 119.22 a

Values within a row marked with different letters (a–c) differ significantly at *p* ≤ 0.05. C—control group; STZ—streptozotocin-injected rats; SPR—rats fed diet with sprats; STZ + SPR—rats injected with streptozotocin and fed diet with sprats; T_4_—thyroxine; T_3_—triiodothyronine; TSH—thyroid-stimulating hormone; GR—glutathione reductase; GPx—glutathione peroxidase; SOD—superoxide dismutase.

**Table 5:** Selected relative gene expression in the thyroid, kidneys, liver, lungs and heart of experimental rats (relative mRNA levels).

Selected Gene	C	STZ	SPR	STZ + SPR
Thyroid				
*Gpx1*	0.94 ± 0.46 ab	1.07 ± 0.68 ab	1.13 ± 0.28 a	0.87 ± 0.45 b
*Gpx3*	0.97 ± 0.12 ab	0.82 ± 0.11 b	1.02 ± 0.20 ab	1.20 ± 0.32 a
*Txnrd1*	0.83 ± 0.30 b	0.79 ± 0.27 b	1.19 ± 0.27 a	1.18 ± 0.41 a
*Dio1*	0.25 ± 0.11 b	0.96 ± 0.47 b	0.93 ± 0.52 b	1.85 ± 1.19 a
*Dio2*	1.34 ± 0.52 a	0.97 ± 0.43 ab	0.86 ± 0.41 b	0.83 ± 0.25 b
*Dio3*	1.46 ± 0.72 a	0.75 ± 0.59 bc	1.20 ± 0.51 ab	0.59 ± 0.21 c
Kidneys				
*Gpx1*	1.13 ± 0.71 a	0.85 ± 0.43 a	0.81 ± 0.46 a	1.22 ± 0.46 a
*Gpx3*	1.55 ± 0.62 a	0.65 ± 0.27 b	0.62 ± 0.21 b	1.18 ± 0.59 a
*Txnrd1*	1.09 ± 0.50 a	1.04 ± 0.28 a	0.85 ± 0.21 a	1.03 ± 0.24 a
*Txnrd2*	0.85 ± 0.53 a	1.18 ± 0.48 a	1.11 ± 0.30 a	0.86 ± 0.31 a
*Dio1*	1.11 ± 0.61 a	0.94 ± 0.52 a	1.12 ± 0.51 a	0.83 ± 0.43 a
Liver				
*Gpx1*	1.44 ± 0.64 a	0.34 ± 0.25 b	0.40 ± 0.33 b	1.82 ± 1.02 a
*Txnrd1*	0.94 ± 0.35 ab	1.57 ± 1.28 a	0.46 ± 0.16 b	1.04 ± 0.45 ab
*Dio1*	1.38 ± 0.53 a	0.43 ± 0.27 b	0.82 ± 0.58 ab	1.36 ± 0.98 a
*Sod1*	1.10 ± 0.26 a	0.50 ± 0.15 b	1.12 ± 0.71 a	0.92 ± 0.46 a
Lungs				
*Gpx1*	0.26 ± 0.13 b	0.62 ± 0.32 b	1.51 ± 0.33 a	1.61 ± 1.06 a
Heart				
*Sod1*	1.06 ± 0.45 a	0.48 ± 0.23 b	0.82 ± 0.49 a	1.2 ± 0.65 ab

Values within a row marked with different letters (a–c) differ significantly at *p* ≤ 0.05. C—control group; STZ—streptozotocin-injected rats; SPR—rats fed diet with sprats; STZ + SPR—rats injected with streptozotocin and fed diet with sprats; *Sod1*—superoxide dismutase 1; *Gpx1*—glutathione peroxidase 1; *Gpx3*—glutathione peroxidase 3; *Txnrd1*—thioredoxin reductase 1; *Txnrd2*—thioredoxin reductase 2; *Dio1*—iodothyronine deiodinase type 1; *Dio2*—iodothyronine deiodinase type 2; *Dio3*—iodothyronine deiodinase type 3.

**Table 6:** Experimental diet composition [g/kg].

Ingredient [g/kg]	C and STZ *	C and STZ **	SPR and STZ + SPR *	SPR and STZ + SPR **
Corn starch	632.486	532.486	629.086	526.816
Casein ^a^	100	200	0 ^a^	87.300 ^a^
Sucrose	100	100	100	100
Soybean oil	70	70	3.9 ^a^	0 ^a^
Dietary fiber	50	50	50	50
Mineral mix ^b^	35	35	35	35
Vitamin mix ^b^	10	10	10	10
Choline	2.5	2.5	2.5	2.5
TBHQ ^c^	0.014	0.014	0.014	0.014
Sprats ^d^	-	-	169.5 ^d^	188.37 ^d^
Mg	-	0.76972 ± 0.05689	-	1.16773 ± 0.03209
Ca	-	4.35666 ± 0.28936	-	6.69202 ± 1.16354
Fe	-	0.08732 ± 0.06266	-	0.04930 ± 0.00668
I	-	0.000076 ± 0.0000021	-	0.000169 ± 0.0000053
Zn	-	0.03812 ± 0.00073	-	0.05340 ± 0.00052
Se	-	0.00014 ± 0.00005	-	0.00041 ± 0.00015
Cu	-	0.00609 ± 0.00017	-	0.00753 ± 0.00135

C—control AIN-93G diet; STZ—AIN-93G diet and streptozotocin injection of rats; SPR-AIN-93G diet supplemented with sprats; STZ + SPR—AIN-93G diet supplemented with sprats and streptozotocin injection of rats. * diets provided 100 g of casein (C-diet) or amount of sprats, which gave 100 g of protein, ** diet with amount of 200 g of casein (C-diet) or lower amount of casein to balance proteins from sprats. ^a^ The protein and fat contents of the SPR diets were balanced to match AIN-93G by replacing part of the casein and soybean oil with an appropriate amount of sprats. ^b^ According to AIN-93G. ^c^ Tert-butylhydroquinone (TBHQ). ^d^ Sprats were added in baked, freeze-dried and ground form; the value reported in the table is expressed as g/kg diet (dry matter basis).

## Data Availability

The raw data supporting the conclusions of this article will be made available by the authors on request. In addition, the data presented in this study are openly available at the University of Agriculture in the Krakow Research Data Repository at DOI 10.15576/REPOURK/2026.1.14.
